# Supercooling: a promising technique for prolonged preservation in solid organ transplantation, and early perspectives in vascularized composite allografts

**DOI:** 10.3389/frtra.2023.1269706

**Published:** 2023-10-23

**Authors:** Yanis Berkane, Justine Hayau, Irina Filz von Reiterdank, Anil Kharga, Laura Charlès, Abele B. Mink van der Molen, J. Henk Coert, Nicolas Bertheuil, Mark A. Randolph, Curtis L. Cetrulo, Alban Longchamp, Alexandre G. Lellouch, Korkut Uygun

**Affiliations:** ^1^ Vascularized Composite Allotransplantation Laboratory, Massachusetts General Hospital, Harvard Medical School, Boston, MA, United States; ^2^ Shriners Children’s Boston, Harvard Medical School, Boston, MA, United States; ^3^ Department of Plastic, Reconstructive and Aesthetic Surgery, Hôpital Sud, CHU Rennes, University of Rennes, Rennes, France; ^4^ MOBIDIC, UMR INSERM 1236, Rennes University Hospital, Rennes, France; ^5^ Division of Plastic Surgery, Lausanne University Hospital, Lausanne, Switzerland; ^6^ Department of Plastic, Reconstructive and Hand Surgery, University Medical Center Utrecht, Utrecht, Netherlands; ^7^ Center for Engineering for Medicine and Surgery, Department of Surgery, Massachusetts General Hospital, Harvard Medical School, Boston, MA, United States; ^8^ Department of Vascular Surgery, Lausanne University Hospital, Lausanne, Switzerland; ^9^ Center for Transplant Sciences, Massachusetts General Hospital, Boston, MA, United States

**Keywords:** supercooling, subzero, organ preservation, VCA, vascularized composite allotransplantation, machine perfusion

## Abstract

*Ex vivo* preservation of transplanted organs is undergoing spectacular advances. Machine perfusion is now used in common practice for abdominal and thoracic organ transportation and preservation, and early results are in favor of substantially improved outcomes. It is based on decreasing ischemia-reperfusion phenomena by providing physiological or sub-physiological conditions until transplantation. Alternatively, supercooling techniques involving static preservation at negative temperatures while avoiding ice formation have shown encouraging results in solid organs. Here, the rationale is to decrease the organ's metabolism and need for oxygen and nutrients, allowing for extended preservation durations. The aim of this work is to review all advances of supercooling in transplantation, browsing the literature for each organ. A specific objective was also to study the initial evidence, the prospects, and potential applications of supercooling preservation in Vascularized Composite Allotransplantation (VCA). This complex entity needs a substantial effort to improve long-term outcomes, marked by chronic rejection. Improving preservation techniques is critical to ensure the favorable evolution of VCAs, and supercooling techniques could greatly participate in these advances.

## 1. Introduction

Transplantation sciences are rapidly evolving, and modern approaches such as machine perfusion are now used routinely for liver (1, 2), kidney (3, 4) heart transplants (5, 6) and to a lesser extent lungs (7, 8). These techniques have progressively supplanted static cold storage and has been shown to be superior in preserving organ function and reducing ischemic injury related to the delay between procurement and revascularization. On the other hand, the increasingly favorable results in solid organ transplantation have prompted reconstructive surgeons to leverage it in addressing their own specific problematics, resulting in the development of Vascularized Composite Allotransplantation (VCA). It consists of transplanting complex tissues composed of vascularized and innervated skin, subcutaneous tissue, fat, muscle, bone, and/or joint. This recent entity started with the first successful forearm allotransplantation in 1998 by Dubernard (9), followed by the first partial face transplantation led by Devauchelle and Lengelé in 2005 in France (10). Since then, a few teams have performed more than 130 upper limb transplants (11, 12), 47 face transplants (13, 14), 6 penis transplants (15, 16), and, more recently, uterus transplants allowing full-term births (17, 18). If the commitment of these pioneering teams has allowed the restoration of some aesthetic and functional units, chronic immune rejection has become a major challenge for the long-term function of these grafts (19, 20). If up to 89% of patients receiving a VCA develop acute rejection episodes, certain immunosuppression protocols allow optimal long-term tolerance (13, 14), up to 20% of patients (19) who have undergone face or upper limb transplants are faced with rejection phenomena in the first 10 years, leading to graft loss (14).

Moreover, current challenges in transplantation research involve improving the organ supply to poorly served areas and enhancing the match between the donor and recipient to allow a better long-term balance between organ function and immunosuppressive drug tolerance. In the specific case of VCAs, this matching concerns not only Human Leukocyte Antigen (HLA), but also skin color, age, and gender. In addition, the functional aspect of these reconstructive surgeries raises an ethical debate concerning the benefit-risk ratio of immunosuppression (21), which is less discussed in solid organ transplantation due to its vital nature. With the increasing longevity of organ transplant recipients, it becomes crucial to address not only the short-term but also the long-term severe side effects that can significantly impact both the quality and quantity of life years. This emphasizes the need for better preservation techniques to increase the pool of available grafts, improve matching, and minimize the impact of an extended *ex-vivo* journey. A few teams have been interested in importing Hypothermic (HMP) and SubNomorthermic (SNMP) machine perfusion from solid organs to VCAs (22–25), to decrease IRI (of the muscle, primarily), and thus improve functional outcomes over time. However, the preliminary results on VCA machine-perfusion were not able to extended preservation beyond a few days (24, 26, 27), as it might be necessary to optimize immune tolerance protocols (>2 days needed) (16).

An attractive alternative that may overcome these current limitations is supercooling preservation, which has been evaluated in solid organ preservation and has recently shown astounding results in allowing human liver preservation for up to 44 h (28). This static storage technique is a simpler approach to prolonged SNMP or even Normothermic Machine Perfusion (NMP) that has also been shown suitable for multi-day liver preservation. The core principle is that cell metabolism decreases with temperature and that reaching sub-zero temperatures while avoiding ice nucleation (29), allows maintaining the organ in an optimal hibernation state for extended preservation: The lower temperature decreases the need for energy expenditure, water loss, mitochondrial respiration and ATP consumption (30). However, each organ and cell type responds differently to preservation conditions due to their different composition, metabolism requirements, and function. Therefore, significant optimization is critical, especially for transposing these recent techniques to VCAs.

The objective here is to review the use of supercooling in solid organ transplantation and to provide insight into the application and challenges of supercooling VCAs in animal models.

## 2. Methods

Literature on Pubmed and Google Scholar databases was reviewed by using the keywords and Boolean operators “Organ AND (Supercooling OR Subzero)”. The search was then repeated by replacing “Organ” with “Liver”, “Kidney”, “Heart”, “Lung”, “Skin”, “Adipo*”, “Bone”, “Nerve” and “VCA”, successively. Additionally, reference lists were manually checked to identify other relevant articles. Non-original articles and articles in languages other than English and French were excluded.

## 3. Literature screening results

The search strategy retrieved 339 total results. Title and abstract screening resulted in including 45 results, covering liver (10), kidney (6), heart (6), lung (6), skin (3), fat/connective tissue (5), nerve (2), bone (3), and VCA (4). Table 1 succinctly displays the main results of the literature review, summarizing citations relevant to each organ and tissue supercooling.

**Table 1:** Results of the literature review on organ and tissue supercooling, presenting relevant citations for each field.

Ref #	Organ/Tissue	Authors	Journal	Year	**Volume**, Pages/electronic pages
(31)	Liver	Ishine et al.	*Cryobiology*	1999	**39**, 271–277
(32)		Ishine et al.	*Cryobiology*	2000	**40**, 84–89
(33)		Rubinsky et al.	*Biochem Biophys Res Commun*	1994	**200**, 732–741
(34)		Takahashi et al.	*Transplant Proc*	2001	**33**, 916–919
(35)		Takahashi et al.	*Transplant Proc*	2000	**32**, 1634–1636
(36)		Monzen et al.	*Biochem Biophys Res Commun*	2005	**337**, 534–539
(37)		Bruinsma et al.	*Nat Protoc*	2015	**10**, 484–494
(38)		Mojoudi et al.	*Am J Transplant*	2023	**23**, 614–1200
(39)		Botea et al.	*Biochem Biophys Res Commun*	2023	**34**, 101485
(40)		Zhao et al.	*Cryobiology*	2022	**106**, 139–147
(41)	Kidney	Tomalty et al.	*Cryo Letters*	2017	**38**, 100–107
(42)		Tomalty et al.	*Cryobiology*	2023	**111**, 113–120
(43)		Sultana et al.	*Transplant Proc*	2018	**50**, 1178–1182
(44)		Jacobsen et al.	*Cryobiology*	1975	**12**, 123–129
(45)		Grundmann et al.	*Eur Surg Res*	1980	**12**, 208–218
(46)		Jacobsen et al.	*Cryobiology*	1979	**16**, 24–34
(47)	Heart	Amir et al.	*J Heart Lung Transplant*	2005	**24**, 1915–1929
(48)		Kato et al.	*Transplantation*	2012	**94**, 473–477
(49)		Seguchi et al.	*Transplant Direct*	2015	**1**, /e33
(50)		Wan et al.	*Biochem Biophys Res Commun*	2018	**496**, 852–857
(51)		Wei et al.	*Cryobiology*	2018	**80**, 161
(52)		Wei et al.	*Cryobiology*	2018	**81**, 225
(53)	Lung	Lee et al.	*Cryobiology*	1995	**32**, 299–305
(54)		Abe et al.	*Ann Thorac Surg*	2006	**82**, 1085–1088
(55)		Okamoto et al.	*J Heart Lung Transplant*	2008	**27**, 1150–1157
(56)		Aguiló et al.	*J Thorac Cardiovasc Surg*	2003	**125**, 907–912
(57)		Kelly	*J Lab Clin Med*	2000	**136**, 427–440
(58)		Omasa et al.	*Transplantation*	2003	**75**, 591–598
(59)	Skin	Kim et al	*Transplant Proc*	2021	**53**, 1756–1761
(60)		Ling et al.	*Eur J Pharm Biopharm*	2023	**189**, 109–121
(61)		Stevens et al.	*Aesthet Surg J*	2023	*Online ahead of print,* *doi: 10.1093/asj/sjad178*
(62)	Fat and connective tissue	MacRae et al.	*Ann Plast Surg*	2004	**52**, 281–282
(63)		Huang et al.	*Cryobiology*	2020	**92**, 67–75
(64)		Williams et al.	*J Exp Biol*	2011	**214**, 1300–1306
(65)		Hittel et al.	*J Exp Biol*	2002	**205**, 1625–1631
(66)		Carey et al.	*Physiol Rev*	2003	**83**, 1153–1181
(67)	Nerve	Babes et al.	*Eur J Neurosci*	2006	**24**, 691–698
(68)		Miller et al.	*Science*	1965	**149**, 74–75
(69)	Bone	Ralis et al.	*J Bone Joint Surg Br*	1989	**71**, 55–57
(70)		Biga et al.	*Anatomy and Physiology*	2023	**1,** Chapter 6.3
(71)		Al Qabbani et al.	*PLoS One*	2023	**18**, e0283922
(72)	VCA	Berkane et al.	*Am J Transplant*	2023	**23**, 614–1200
(73)		Filz von Reiterdank et al.	*Cryobiology*	2022	**109**, 19–20
(74)		Nakagawa et al.	*J Orthop Sci*	1998	**3**, 156–162
(75)		Zhang et al.	*Am J Transplant*	2023	**23**, 614–1200

## 4. Supercooling principles to extend organ preservation

Organ mitochondrial oxidative metabolism has been described to be reduced by half for every 10°C drop in temperature (76). Static cold storage (SCS) has utilized these principles for decades by storing organs at 4°C, mostly on ice. However, if this process allows for decreasing ischemic injuries, storage at 4°C does not completely arrest cellular metabolism and limits the total preservation duration. Recent developments in machine-perfusion techniques have aimed to decrease these injuries by oxygenating the organ while being cooled: Hypothermic Oxygenated Machine Perfusion (HMP) has shown interesting results in liver (2) and kidney (77) transplantation. Sub-zero temperatures have the potential to further decrease cell metabolism. Intracellular ice formation is one of the main causes of cell death during sub-zero preservation (78). Ice formation is a stochastic phenomenon and increases in lower temperatures and larger tissue volumes. Different approaches coexist in these temperature ranges.

Partial freezing consists of using ice nucleators to control and limit ice formation and cryoprotective agents (CPAs) to maintain an unfrozen liquid phase (29). This preservation method allows for storage at −10 to −15°C and has shown positive outcomes in livers (79). Alternatively, Vitrification is a technique that involves rapidly cooling organs to achieve a stable, ice-free state with a glass-like consistency (80). However, the ice-nucleation issue arises when these organs are warmed back to normal temperatures. The formation of ice crystals has been a major limitation during vitrified organ recovery if the rewarming process is too slow. In addition, another risk is of cracking due to thermal stress if the rewarming is not done uniformly. Several teams have been working on fast and uniform rewarming of vitrified organs using nanoparticles, with promising results in kidneys (81).

**Figure 1:**
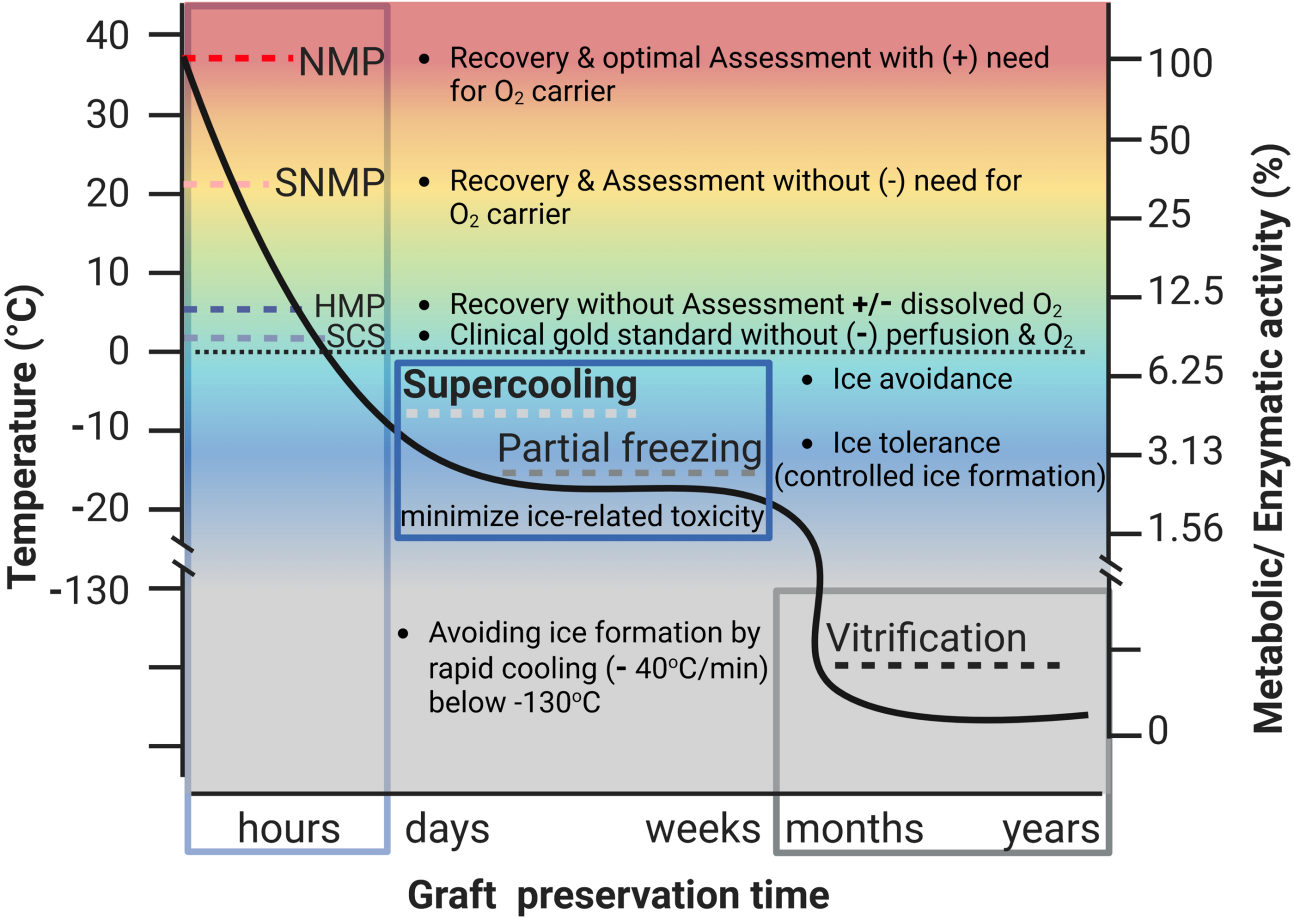
Organ preservation methods based on the temperature: dynamic preservation techniques include normothermic machine perfusion (NMP) at 37°C, Sub-normothermic machine perfusion (SNMP) at room temperature (19–23°C), and hypothermic machine perfusion (HMP) at 4°C. In Static Cold Storage (SCS), the graft is preserved at ice-cold temperature (closer but slightly above 0°C) and still represents a clinical gold standard until now. Supercooling consists of sub-zero preservation at −4°C to −10°C in a liquid state without ice formation, whereas partial freezing comprises sub-zero preservation at −10°C to −15°C with controlled extra-cellular and intra-vascular ice formation while preventing intra-cellular ice formation using specific cryoprotective agents (CPAs). Vitrification is rapid cooling at a rate of −40°C/min below −130°C using concentrated CPA avoiding ice formation, which results in a glass-like state with metabolic activity close to zero helping in long-term bio-banking. This latest technique is currently limited to cells and small organs due to limitations with large volume rapid rewarming needed for recovery post-preservation phase. Several teams have described nanoparticles as a novel technology allowing gradual and uniform rewarming of the vitrified organ. Massive improvements in this field are expected in the near future.

In contrast to partial freezing, supercooling aims to achieve preservation at below 0°C temperatures while avoiding ice nucleation (Figure 1) (28). The prevention of ice formation must be done both in the extracellular and intracellular spaces. For this purpose, various CPAs are used. This is the most critical difference with partial freezing protocols that aim to provoke controlled ice nucleation (82). CPAs have inherent properties that allow them to remain in a liquid state at sub-zero temperatures, in addition to increasing the osmolarity of the intracellular and extracellular liquid compartments, decreasing their freezing point (83). Some authors have described 3-O-Methyl-Glucose (84) as a nonmetabolizable glucose that can prevent intracellular freezing. Several teams have used it in their supercooling protocol since then. Dimethyl sulfoxide (DMSO) has also been widely used for various applications in cryobiology but seems to provoke cell toxicity (85). As for extracellular CPAs, needed to prevent the intravascular compartment and the surrounding media from freezing, most of the preservation protocols in solid organs have been using polyols and polyethers such as glycerol and polyethylene glycol (PEG) (86–41). Nature-inspired molecules like Trehalose, a complex sugar used by plants to avoid freezing, have also been experimented with (88, 89). Different protocols were tested for various organs, involving diverse processes.

### 4.1. Liver

Rubinsky et al. performed one of the first studies in rat livers in 1999 (31), comparing freezing and supercooling. The authors showed that ice nucleation was propagating along the vasculature, starting in contact with the endothelial cells. Moreover, they showed that both preservation techniques allowed for preserving the hepatic architecture but that freezing was associated with extensive endothelial injuries. None of their freezing protocols allowed for preserving the sinusoïdal endothelial cell lining. This team later performed liver transplantation following high subzero preservation mimicking freeze-tolerant animal physiology (32). This confirmed that microcirculation failure was one of the critical limitations to address (33). In contrast, they showed better preservation of the capillary endothelial lining in the supercooled livers, showing promising potential of this technique. Takahashi (34) explored high-pressure supercooling, hypothesizing that ice nucleation can be avoided by increasing the hydrostatic pressure without provoking liver tissue injuries. They were able to supercool rat livers by using conventional University of Wisconsin (UW) media in a pressure chamber (10–70 MPa), but the transplanted livers were not viable as proven by the early death of the liver recipients after more than 60 min of preservation. They still achieved optimization with successful transplants after 5 h of supercooling (−2°C) by adapting the hydrostatic pressure to 30 MPa (35) and hypothesized that liver supercooling could be performed without CPAs by using pressure chambers. However, their work consisted in short (1–5 h) preservation, and extended durations should be tested for more relevance. Monzen et al. (36) later studied organ supercooling in an engineered refrigerator with electrostatic voltage. They showed that cytolysis was lower in livers supercooled at −4°C in UW compared to SCS. However, this work lacked normothermic reperfusion, which can unveil severe injuries following cold preservation.

**Figure 2:**
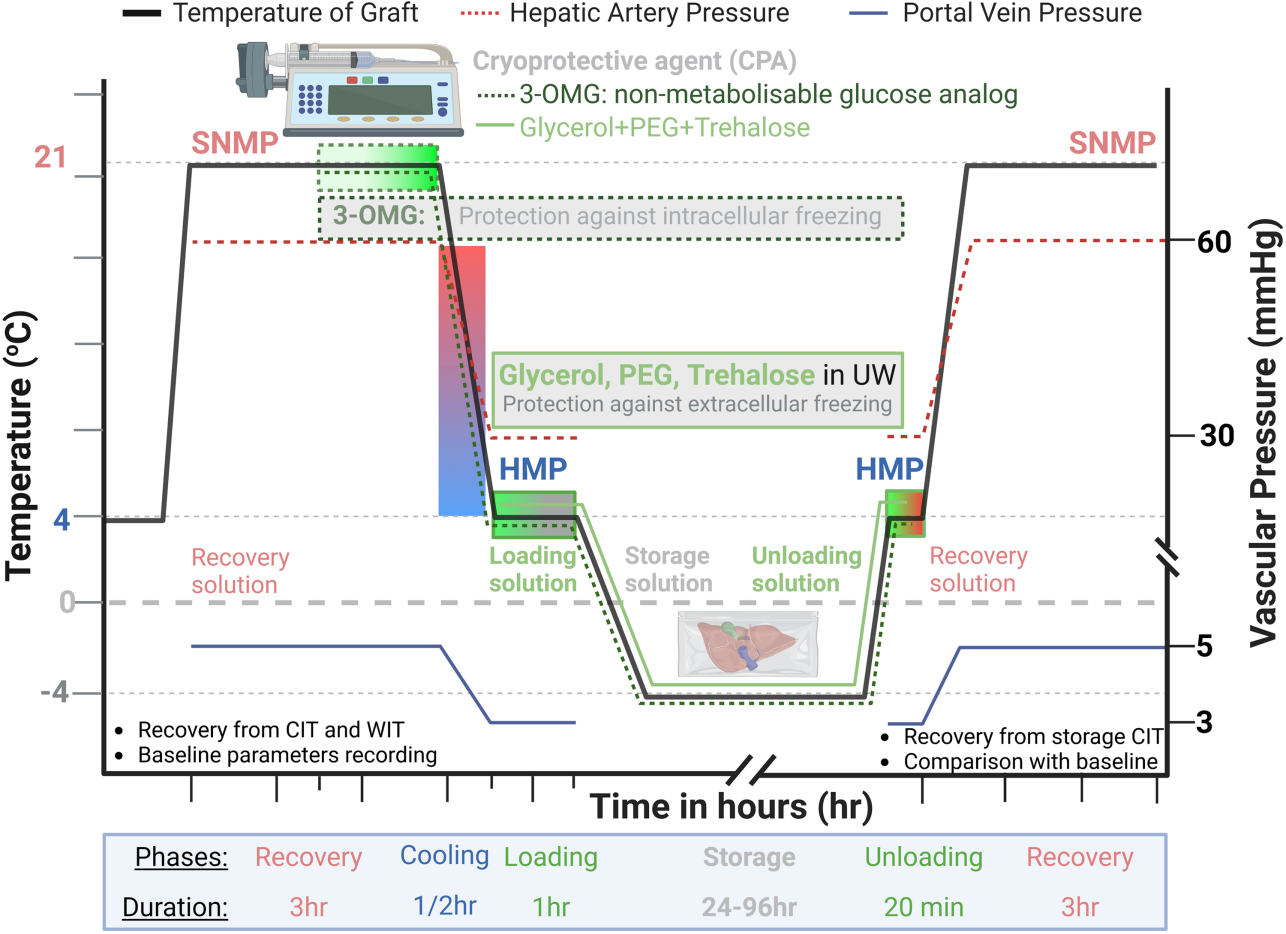
Supercooling protocol of human liver: liver after supercooling storage is recovered using Sub-normothermic machine perfusion (SNMP) for 3 h at room temperature (21°C) using a physiologic recovery solution. Starting on the SNMP mid-phase, 3-OMG (3-O-methyl-glucose), a non-metabolizable glucose analog, is infused slowly into the graft to prevent intracellular freezing. After SNMP, the graft is cooled for half an hour to 4°C for loading cryoprotective agents [CPA: Glycerol, PEG (Polyethylene Glycol), and Trehalose] using University of Wisconsin (UW) preservative solution as a base. This loading lasts for 1hr using oxygenated hypothermic machine preservation (HMP) to allow a uniform distribution of CPA, preventing extra-cellular and intravascular freezing while the graft is stored at sub-zero temperatures (−4°C). After extended storage, the graft is recovered first by unloading CPA out of the vascular tree using an unloading solution during HMP, followed by 3 h of SNMP with a physiological recovery solution. The portal vein and hepatic artery pressures are kept around 5 and 60 mmHg during SNMP and 3 and 30 mmHg during HMP, respectively. These values take into account the increased viscosity and shear stress at lower temperatures. The perfusion parameters are assessed pre and post-supercooling and compared to evaluate graft function.

Our lab published promising results in 2014 (37, 86), describing for the first time a complete protocol for liver supercooling using a standard refrigerator. This recent work demonstrated optimizing a CPA loading protocol using subnormothermic machine perfusion, achieving storage for 96 h prior to successful transplantation. This represents a 3-fold increase in comparison with the usual preservation duration. SNMP has a major advantage over previous methods: For proper intracellular loading of the non-metabolized glucose (3-OMG), the organ needs to be active (i.e., in subnormal temperature), while extracellular freezing was prevented by loading the UW solution with PEG, allowing supercooling at −6°C. Additionally, the air/media interface was eliminated by sealing the supercooling media with an immersible phase. This work was the first complete liver supercooling protocol precisely described (86, 89), allowing for adequate reproducibility and scaling to large animals and/or clinics (Figure 2). This protocol was later upscaled to human livers (28) with successful preservation for 27 h assessed by whole blood NMP as a transplantation simulation. The human liver supercooling protocol included adding trehalose and glycerol for further extra and intracellular cryoprotection, respectively. Finally, our latest work showed liver viability after 3 days of supercooling preservation and SNMP recovery (38). This work in liver has been used as a basis for modern supercooling techniques applied to VCA (72, 73).

Finally, the work developed by Rubinsky and his team on isochoric (constant volume) supercooling showed successful supercooling in porcine livers for up to 48 h at −2°C (39), taking up the challenge of subzero non-freezing state in a large organ. Their team also developed a system for cryomicroscopy, making it possible to study biological systems at subzero temperatures and understand the cell behavior toward ice formation (40).

### 4.2. Kidney

Kidney grafts for transplantation are typically stored on ice at 4°C (SCS). In a multi-center randomized controlled study, hypothermic (4°C) machine perfusion reduced ischemia-reperfusion injury compared to SCS (90, 91) and was cost-effective (92). While machine perfusion strategies have gained wide interest in kidney transplantation, these preservation strategies using FDA-approved devices are mostly limited to a few hours, with maximum perfusion duration reported for up to 2 days. Of importance, success in extending the viable preservation time beyond the current limits will contribute to global organ sharing and increased kidney availability. In the following section, we will review the available evidence of sub-zero non-freezing preservation in kidneys.

*In vitro*, immortalized human proximal-tubule epithelial cells stored at −6°C for up to 6 days with antifreeze proteins or cryostasis storage solution showed decreased viability, increased lactate dehydrogenase (LDH) release, and apoptosis compared to 4°C storage in UW solution. This suggests that this kidney cell type might be prone to chilling injury and that the addition of antifreeze proteins and cryostasis may not be effective for increasing storage times at subzero temperatures (42). However, it was demonstrated that preservation of neonatal rat kidneys at −2°C for 48 h in histidine-tryptophan-ketoglutarate (HTK) solution at −2°C reduced histological damage (cortex and medulla) compared to 24 or 4°C (43). Consistently, rat kidneys stored in cryostasis storage solution with or without the addition of a hyperactive insect antifreeze protein for 48 h and 72 h, respectively, demonstrated similar viability (MTT reduction assays, caspase-3 and calpain activity) compared to kidneys stored for 24 h in UW (41).

Prior to autotransplantation, Jacobsen et al. (44) stored rabbit kidneys at −6°C for 1 h with different concentrations of CPA (glycerol or propylene glycol) in three vehicle solutions with varying potassium levels and the presence of mannitol. Interestingly, they observed that up to 3 Molar (M) glycerol or propylene glycol in the normokalemic vehicle solution was well tolerated, but not in the hyperkalemic solutions. Using 4M propylene glycol in either solution resulted in severe damage with no survival post-transplant. The findings suggest that propylene glycol is toxic above 3M, suggesting that a combination of propylene glycol and glycerol should be considered. Somehow consistently, immediate renal function post-canine kidney autotransplantation (p-aminohippuric acid and inulin clearances) was improved by preservation (24 h) in a hyperosmolar perfusate at −2°C compared to 4°C Collins solution (45).

Finally, while cooling of rabbit kidneys with 3M glycerol at −5°C for 50 min resulted in 80% survival at 20 days post-transplant, storage at −80°C was associated with a marked increase in vascular resistance after thawing, and none of such frozen kidneys functioned after transplantation (46). Differences with the liver, with regard to the effects of preservation at sub-zero temperatures, may be linked to the different cell types, volumes, and, above all, vascularization: the glomerular capillaries of the kidney differ from the sinusoidal capillaries of the liver, which are open to the perisinusoidal space. The difference in permeability between these two capillary exchange systems suggests a different passage of CPAs into the interstitial tissues, suggesting dissimilar effects. Altogether, these studies suggest that kidney subzero storage is feasible. However, significant progress needs to be made in the combination of CPA and base media (with particular attention to electrolytes), loading and unloading strategies, and defining the optimal timing for storage and each of the perfusion phases.

### 4.3. Heart

Similarly to other solid organs, the storage solutions were supplemented with antifreeze proteins (AFPs) with cryoprotective properties. Rat hearts stored in UW solution containing antifreeze proteins I and III at −1.3°C for 21 h improved left ventricular function and lowered apoptosis after heterotopic transplantation compared to SCS (47). Of interest, some authors immersed rat hearts in a UW solution associated with a variable magnetic field to induce a supercooled state without using CPAs at −3.0°C prior to 24 h storage, followed by 120 min of reperfusion. Subzero preservation improved post-ischemic left ventricular function, increased myocardial ATP level, and decreased tissue edema (48). Using similar methods, porcine hearts stored at −3°C had higher ATP compared to controls (21.06 ± 5.87 μmol/g vs. 5.96 ± 3.41 μmol/g; *P* < 0.05). The accumulated lactate concentration was significantly lower in the subzero group than in the conventional group (6.58 ± 2.28 μmol/g vs. 11.15 ± 3.74 μmol/g; *P* < 0.05). The Flameng score, an index of ultrastructural changes in mitochondria, was significantly lower in the subzero group than in the conventional group (1.28 ± 0.40 vs. 2.73 ± 0.30; *P* < 0.05). Altogether, subzero ice-free preservation using a variable magnetic field suppressed anaerobic metabolism and was associated with myocardial protection in both rat and porcine hearts (49). However, future studies would need to show the effect of the magnetic field and its benefit compared to CPAs or isochoric freezing, as no comparative analyses were carried out in the presented studies.

Interestingly in rats, some authors have used isochoric (constant volume) storage at subfreezing temperatures without any CPA for one hour at 0°C (atmospheric pressure -0. 1 MPa), −4°C (41 MPa), −6°C (60 MPa) and −8°C (78 MPa). Lowering the storage temperature reduced metabolism and improved storage quality by decreasing the occurrence of interstitial edema, suggesting an effect on vascular permeability. It also limited the appearance of cell damage and prevented the increase in pressure for storage temperatures down to −6°C. This suggests that preservation is improved at −4°C with low to moderate pressures. Tissue damage was observed at lower temperatures (−6°C or lower), accompanying further pressure elevation. Further optimization between temperature and pressure is needed to mitigate the adverse effects of high pressure while preserving the advantageous impact of lower temperatures (50). Finally, in mice, storage at −8°C for 96 h reduced ischemia-reperfusion injuries, oxidative stress, and apoptosis of myocardial cells, which resulted in improved survival post-transplant compared to storage at 4°C in UW (87). Their study showed a decrease in myocyte metabolism. As muscle tissue is highly sensitive to ischemia, these findings are in favor of the applicability of supercooling to VCA by showing relevant outcomes in striated muscle.

Recent organ preservation solutions, including XT-ViVo (X-therma, Richmond, CA) (51, 52), have achieved FDA clearance with Breakthrough Device Designation due to their remarkable potential to extend preservation duration up to 7 days. These advancements in diminishing the CPA toxicity by using nature-inspired antifreeze molecules hold significant promise for extending the viability of sensitive organs such as heart or VCA before transplantation.

### 4.4. Lung

Current lung preservation is limited to 8 h at 4°C (93). Extension of this preservation time was shown above zero, at 37°C for 12 h using *ex vivo* lung perfusion (EVLP) (94), at 10°C for 36 h using SCS in pigs and humans (95), and at 10°C with 4 h cycles of 37°C EVLP for 72 h using a combination of SCS and EVLP in pigs (96). Below zero, Lee et al. (53) analyzed the cooling rate of the different lung tissues since this organ's *in vivo* composition is 80% of air. They showed a faster cooling rate in the subpleural areas when compared to solid organs. The heat conductivity was found to be lower in the lung du to air insulation. Later, the specific potential of supercooling as a lung preservation method was suggested by Abe et al., showing normal histological appearance in −5°C stored lungs in contrast to lungs stored at 4°C (54). Additionally, lower levels of single-stranded DNA were found. Whilst this study was performed on human lung tissue, no functional assessment was performed after storage. In a rat study, Okamoto et al. (55) compared 17 h storage at −2°C without additional CPAs to SCS at 4°C and fresh controls. Whilst reperfusion only lasted for 60 min, endothelial lining and deterioration of perfusion parameters (tidal volume, oxygen levels, arterial pressure), as well as lower ATP levels, were comparable between the fresh and the supercooled lungs. Another study investigated 72 h storage at 0°C using ethanol as CPA and compared this to storage at 10°C for 24 h and 72 h and fresh controls (56). Whilst the preserved groups showed higher injury levels than fresh controls during the 60-min reperfusion, lung function assessed based on either effluent PO2, peak airway pressure, or mean arterial pulmonary pressure was better in the 0°C group than both 10°C groups. Paradoxically, lung cell structure—based on the percentage of viable cells using the trypan blue exclusion method—was better preserved in the 24 h group compared to both 72 h groups.

Besides ATP- and NADPH-related pathways that lead to oxidative stress, cessation of flow has also been shown to activate NADPH oxidase, which in turn generates ROS and activates nitric oxide (NO). This pathway is also known as “mechanotransduction”. Normally, NO causes vasodilation and ROS neovascularization. In lung transplantation, however, overproduction of NO and ROS has been shown to cause oxidative injury and activation of proteins that drive inflammation of cell death (97). As supercooling is a static preservation method, this pathway remains relevant.

Similarly to the muscle component of VCAs (98), weight gain during preservation remains a major limitation for lungs (99): It reflects interstitial edema that is a consequence of abnormal vascular permeability and potential endothelial injuries. Interstitial edema also increases microvasculature compression, reflected in higher vascular resistance, closing the vicious circle. Due to this, colloid storage solutions, such as Euro-Collins and UW, have shown superiority in lung preservation (100, 101) Moreover, Steen solution, a perfusate developed for lung perfusion (102) has shown good results in translating to use in VCA perfusion—with a modified composition (24)—including as part of supercooling protocols (73, 103). Furthermore, it has been suggested that hyperkalemic storage solutions result in significant pulmonary vasoconstriction (104), which causes less uniform solution distribution and compromised preservation (105), and poor lung function after transplantation as a result. Alternative solutions (57) and additives such as glycine that have been shown to reduce oxidative injury and apoptosis (58) have been researched in the context of SCS. More specifically, for supercooling, only ethanol as a lung CPA has been investigated (56). Future supercooling protocols can benefit from including learnings of SCS studies in preservation cocktail development, with the potential of pushing the boundaries of static lung preservation time.

### 4.5. Skin, fat, nerve, and connective tissue

In 2021, Kim et al. (59) demonstrated in skin grafts that SCS led to major degenerative changes after 3 days, including cell edema and cytoplasmic blebbing, compared to supercooling. After 7 days, they found a partially detached epidermal-dermal junction in the SCS group, with complete detachment at 14 days, whereas supercooled skin grafts showed physiological skin architecture. These results were confirmed with cell viability assessments showing significantly higher cell survival in the supercooled group. Moreover, since transplanted skin vasculature is a critical factor for successful engraftment (106), they used HUVECs as a component model of skin vasculature to assess the effects of supercooling on their viability. Cell viability was increased, and structural deformation was reduced when compared to SCS. However, Ling et al. highlighted the importance of the storage media by comparing skin viability following cryopreservation in DMSO and glycerin, and without CPA, for 7 days at −20°C (60). If their study did not focus on preventing ice nucleation in supercooling temperatures, it highlights again the critical role of the CPA in ensuring cell homeostasis during subzero storage. Finally, Stevens et al. (61) proved that supercooled skin during cryolipolysis treatment induced Heat Shock Protein 70 expression, which is known as a thermal stress protector transcription factor.

A few authors described adipose tissue preservation using sub-zero storage. MacRae et al. (62) showed 75% adipocyte survivability following 8 days of simple freezing at −20°C, with no specific media. These results suggest that ice nucleation has minor toxicity in adipocytes. One theory is that intracellular lipids, which represent most of the cell volume, undergo less volume change with freezing as compared with water-filled cells (62). Huang et al. (63). achieved stable (7 days) preservation at temperatures as low as −16°C with high viability following recovery. Their CPA solution was based on UW, PEG, and 3-OMG, similar to the liver. The recovery was performed at 4°C then 37°C and cell metabolic activity was assessed in addition to cell membrane integrity, showing up to 95% of viable cells. In addition, the cell stemness potential was preserved, consistent with natural Hibernators such as arctic ground squirrels (64) show no major difference in metabolic gene expression during 2°C and −10°C hibernation, suggesting a relatively preserved metabolism in adipose tissue (65) at below-zero temperatures. Carey and al. showed in 2003 that hibernating animals preferentially catabolize lipid stores (66). These studies suggest the great potential of the adipose tissue for supercooling and tend to reassure about this component of VCAs during subzero nonfreezing preservation.

Animal models have shown that nerves can adapt to cold temperatures (67) by adjusting calcium ion flux. However, to date, only one study has investigated nerve conduction behavior during supercooling. Miller used excised peripheral nerves from Alaskan mammal species to study nerve activity depending on the subzero temperatures (68). With no CPAs used at the time of this study (1965), he found action potentials at temperatures as low as −4.5°C in muskrat and −6°C in beaver tibial nerves using metal chambers and Locke's solution. The amplitude of the action potentials was increased during the spontaneous rewarming of the nerve's environment, further showing adaptation to the supercooling state. As the functionality and thus success of VCAs heavily depends on their sensibility, the field would benefit greatly from a more thorough understanding of the effect of supercooled storage on Wallerian degeneration, Schwann cell survival, and eventually nerve function by assessing nerve conduction in a whole neuromuscular system including the myoneural junction and myocytes. Long-term outcomes after transplantation hold significant importance as they directly influence functional outcomes, regardless of graft survival.

To date, no study has studied supercooled connective tissue. However, Gainaru et al. found (107) that collagen and elastin present temperature-linked relaxation but no defect diffusion (i.e., no change in their properties and behavior).

### 4.6. Bone and stem cells

Bone and joints are critical components for upper limb VCA, since they allow its function. The literature regarding supercooling these tissue remains poor so far. Some authors showed that joints undergo mechanical changes after undergoing sub-zero storage (69). Supercooling of the isolated bone has not been studied yet, but two components should be separately considered: The bone mineral structure is made of a strong matrix containing relatively few cells and calcified fibers (1/3 organic made of collagen, 2/3 inorganic made of calcium phosphate salts including hydroxyapatite (70). Despite the robustness of this matrix, research in decellularized bone has shown a three-fold lower flexural elasticity when compared with native bone (71). These results emphasize the importance of preserving the osteoblasts, osteocytes, osteoclasts, and osteogenic stem cells during preservation in order to ensure adequate function. Among those, stem cell integrity seems to be a critical outcome. In 2002, Matsumoto et al. (108) studied supercooling of human blood stem cells, showing optimal integrity following sub-zero nonfreezing storage at −2°C in UW solution with no CPAs. The cell viability at 72 h was found to be up to 90%, which was significantly higher than static cold stored (4°C) specimens. Cooling was not tested below −2°C to ensure non-freezing. In mesenchymal stem cells, Lauterboeck et al. found that DMSO decreased the temperature threshold to reach 50% ice nucleation from −8.5°C to −11°C (109). Further work should study the effects of cryoprotective agents on these stromal cells, in addition to bone and cartilage resident cells.

### 4.7. Perspectives in vascularized composite allotransplants

VCAs are a more recent field, and the research literature is currently flourishing in topics imported from solid organ transplantation. So far, a few teams only used HMP, SNMP, and NMP techniques. Kruit et al. have shown pioneering work with up to 36 h preservation of musculocutaneous VCA in a pig model using HMP (26, 110). Pig hindlimbs were also successfully preserved for 24 h using SNMP before transplantation, as shown in our preliminary study (111). However, to bridge the gap and allow for multi-day preservation prior to transplantation, applying supercooling principles inspired by solid-organ outcomes could be a promising approach. The rationale behind this objective is, above all, to improve long-term outcomes of VCA procedures. The major challenge is targeting immune rejection. Some transplant centers have described the successful induction of immune chimerism as a promising solution in human renal transplantation (112, 113). This breakthrough led to the first descriptions of VCA immune tolerance protocols in preclinical studies (74). Achieving VCA immune tolerance via mixed chimerism could eliminate the need for permanent immunosuppression, eradicating its side effects such as infections, chronic renal failure, cardiovascular aging, and secondary malignancy (114, 115). However, these innovative protocols require time to prepare the recipient to ensure the best results. This poses a logistical concern since organ donation is not predictable or easily planned. One of the solutions to make these techniques more reliable and simplify their implementation would be to extend the preservation of VCAs during the necessary conditioning: If conditioning the recipient for immune tolerance is adapted to kidney transplants with living donors, this is not applicable to VCAs, since the allograft can only be procured after the confirmed death of the donor. This starts a race against time ensues to perform the transplantation below the ischemia threshold, which varies depending on the tissue: Skin and connective tissue can undergo up to 12 h of cold ischemia, vs. 6–8 h for the muscle, with substantial damage appearing after 2 h only. Bone and nerves can resist for up to 24 h of cold ischemia (116, 117). Following an extended ischemia period, the reperfusion at normothermic temperature with the recipient's blood will induce important inflammation, cytokine release, and acute immune response leading to cell damage and antigen release: these are known as ischemia-reperfusion injuries (IRI) (116–118). Therefore, the muscle tissue is the limiting factor, imposing a replantation as early as 6 to 8 h following the procurement. To ensure the clinical feasibility of immune tolerance through mixed chimerism, preservation of VCAs for several days is, therefore, necessary (74). It, therefore, seems judicious to consider the optimization of supercooling techniques in VCAs with this purpose in mind.

**Figure 3:**
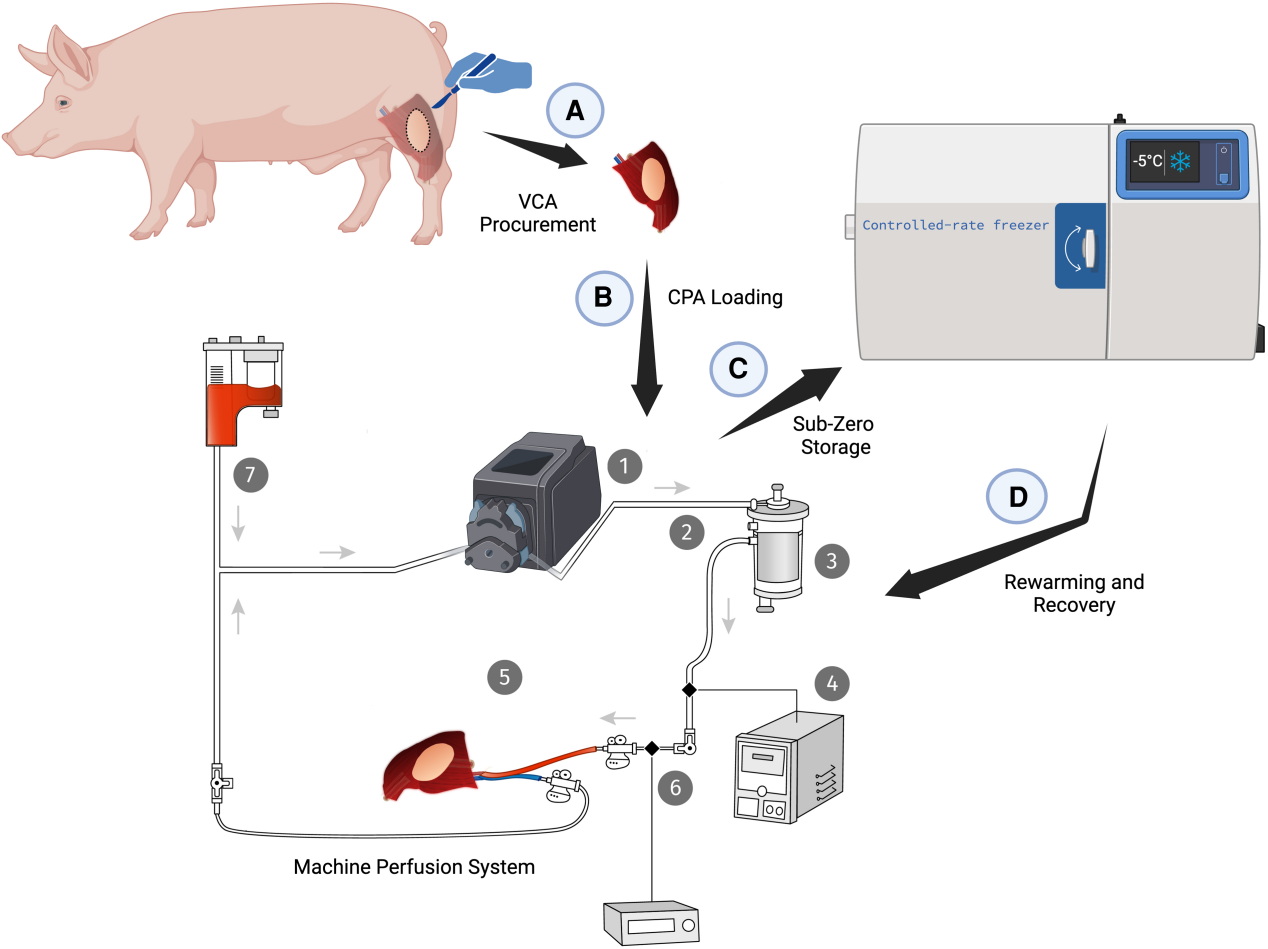
Supercooling of porcine VCA using subNormothermic machine perfusion (SNMP) (**A**), cryoprotective agent (CPA) loading (**B**), storage in a sub-zero non-freezing cocktail at −5°C for 48 h (**C**), and recovery using SNMP(**D**). Machine perfusion system: 1: Peristaltic pump; 2: Silicon tubing; 3: Hollow fiber membrane oxygenator; 4: Cooling system; 5: VCA; 6: Pressure sensor and monitor; 7: Perfusate solution reservoir. Acronyms: VCA, vascularized composite allotransplantation; CPA, cryoprotective agent.

Work from Nakagawa et al. (119) assessed supercooling of rat hindlimbs at −1°C for 72 h prior to transplantation. No CPAs were used in their study published in 1997. The follow-up period of 7 days allowed for relevant histologic assessment of the muscle and bone. Their results showed that supercooling permitted better bone viability with osteoblastic activity after 8 h of −1°C storage when compared to regular cold storage at 4°C. Moreover, vascular patency and histological preservation of the muscle was improved after 72 h of subzero preservation. The skin only showed edema and inflammation in both conditions, with mild improvement by supercooling. However, the bone marrow showed no viable cells above 12 h of preservation in both supercooled and control groups. This last result suggests that reaching −1°C seemed to be insufficient to ensure adequate VCA preservation and that further research is needed to achieve sufficient metabolic depression. Preliminary results from our team in a rat hindlimb model (73) demonstrated successful supercooling during 24 h using a CPA cocktail solution composed of trehalose, glycerol, and PEG, as used in the liver. After 3-OMG loading using SNMP, the limbs were successfully supercooled for 24 h and assessed during SNMP recovery. The major challenge remained weight gain, which was higher than 25% in all supercooled groups, and considered the earliest parameter for VCA preservation failure in machine-perfusion techniques (98). However, no histological differences were found with SCS controls, probably because of the absence of normothermic reperfusion revealing ischemia-reperfusion injuries. This limitation was circumvented by Dr. Brandacher's lab, which performed 72 h supercooling of rat forelimbs before orthotopic transplantation (75). This study allowed for the assessment of supercooled rodent VCA by transplantation and a 4-week follow-up period. To scale up these preliminary rodent results, we recently performed another study in pig hindlimbs (72) using a similar protocol modified with incremental intracellular CPA loading. After 48 h of supercooling preservation, the VCA underwent SNMP recovery, followed by 2 h of NMP using autologous whole blood (Figure 3). The purpose of the SNMP phase was to recover a sub-physiological metabolism following 48 h of cell “hibernation”. Indeed, SNMP has proven to successfully recover static cold stored limbs, progressively reaching vascular resistance, potassium, and lactate values similar to fresh limbs (24). This feature allows better clinical outcomes in SNMP-recovered transplanted limbs when compared with cold storage with no recovery (24). In contrast, the NMP phase in our protocol was designed to assess IRI by recreating a normothermic and physiological setting by perfusing the limb with whole blood. The post-reperfusion injuries were found mild in the supercooled porcine limbs when compared with SCS. The vascular resistance seems to be better preserved with supercooling, but further optimization is needed before *in vivo* application. Overall, these preliminary evidences suggest that VCA supercooling is a promising approach to extending preservation, but with a challenge increased by the muscle component, highly sensitive to ischemia and osmotic shock. Finally, it seems critical to implement nerve conduction assessment of VCAs to address the functional dimension of these reconstructive transplantations. Whilst electroneuromyography can be implemented before, during, and after VCA supercooling, long-term assessment after transplantation will be indispensable to ensure the representability of the results during the supercooling phases and to assess the functional outcomes of these grafts.

## 5. Conclusion

Subzero nonfreezing techniques have shown the potential to expand preservation time in organ transplantation. This literature review of the current evidence suggest that this promising technique may allow for preservation durations long enough to facilitate tolerance induction, successful HLA matching, improved organ availability and supply, and customization to the recipient's needs. Few teams are actively trying to apply supercooling to VCAs, for which storage durations should aim of at least 48 h as this is the current minimal time needed for immune tolerance protocols. This target would also be sufficient to address logistical challenges. Future studies should focus on human organs, and VCA research should include long-term outcomes of nerve function following extended storage durations to allow relevant translation to the clinic.
